# Multiple Types of Novel Enteric Bopiviruses (*Picornaviridae*) with the Possibility of Interspecies Transmission Identified from Cloven-Hoofed Domestic Livestock (Ovine, Caprine and Bovine) in Hungary

**DOI:** 10.3390/v13010066

**Published:** 2021-01-06

**Authors:** Zoltán László, Péter Pankovics, Gábor Reuter, Attila Cságola, Ádám Bálint, Mihály Albert, Ákos Boros

**Affiliations:** 1 Department of Medical Microbiology and Immunology, Medical School, University of Pécs, H-7624 Pécs, Hungary; ifj.laszlozoltan@gmail.com (Z.L.); pankovics.peter@pte.hu (P.P.); reuter.gabor@pte.hu (G.R.); 2 Ceva Phylaxia Ltd., H-1107 Budapest, Hungary; attila.csagola@ceva.com (A.C.); mihaly.albert@ceva.com (M.A.); 3 Department of Virology, National Food Chain Safety Office Veterinary Diagnostic Directorate, H-1143 Budapest, Hungary; BalintAd@nebih.gov.hu

**Keywords:** picornavirus, IRES, bopivirus, livestock, epidemiology, ovine, caprine, bovine, RT-PCR, phylogenetics

## Abstract

Most picornaviruses of the family *Picornaviridae* are relatively well known, but there are certain “neglected” genera like *Bopivirus*, containing a single uncharacterised sequence (bopivirus A1, KM589358) with very limited background information. In this study, three novel picornaviruses provisionally called ovipi-, gopi- and bopivirus/Hun (MW298057-MW298059) from enteric samples of asymptomatic ovine, caprine and bovine respectively, were determined using RT-PCR and dye-terminator sequencing techniques. These monophyletic viruses share the same type II-like IRES, NPGP-type 2A, similar genome layout (4-3-4) and *cre*-localisations. Culture attempts of the study viruses, using six different cell lines, yielded no evidence of viral growth in vitro. Genomic and phylogenetic analyses show that bopivirus/Hun of bovine belongs to the species *Bopivirus A*, while the closely related ovine-origin ovipi- and caprine-origin gopivirus could belong to a novel species “Bopivirus B” in the genus *Bopivirus*. Epidemiological investigation of N = 269 faecal samples of livestock (ovine, caprine, bovine, swine and rabbit) from different farms in Hungary showed that bopiviruses were most prevalent among <12-month-old ovine, caprine and bovine, but undetectable in swine and rabbit. VP1 capsid-based phylogenetic analyses revealed the presence of multiple lineages/genotypes, including closely related ovine/caprine strains, suggesting the possibility of ovine–caprine interspecies transmission of certain bopiviruses.

## 1. Introduction

Picornaviruses (PVs) of the family *Picornaviridae* are a diverse group of small, non-enveloped viruses with a positive-sense single-stranded RNA genome. The ca. 6.7–10.1 kilobase (kb) long, modularly organised PV genome predominantly contains a single viral polyprotein-encoding open reading frame (ORF) flanked by 5′ and 3′ untranslated regions (UTRs) and a 3′ terminal poly(A)-tail [1,2]. Just prior to the start codon, the 5′UTRs of PVs contain one of at least five types of highly structured internal ribosomal entry sites (IRES), which is responsible for the cap-independent translation initiation of the viral polyprotein [1,3]. The cis-acting replication elements (*cre*) are generally built up from a single stem-loop which includes the conserved AAAC motif, and its location in the genome could be conserved among the members of the same genus [4,5]. The ORF is usually organised in a general layout of (L)-**P1**:VP0 (or VP4-VP2)-VP3-VP1-**P2**:2A-2B-2C-**P3**:3A-3B-3C-3D (L = Leader protein), although slight differences like the presence of intergenic IRES between P1 and P2 or region-duplications are also described [1,2,6]. 

From the capsid peptides presented in the P1 genomic region, the VP1 is known to be the most immunodominant peptide predominantly responsible for the sero/genotype of the virus and also frequently used for the in-silico genotype-determination of picornaviruses, e.g., entero-, sapelo-, cardio- or erbo-viruses [7,8,9,10]. The 3′ UTR is ranged between 25 and 825 nucleotides (nt) without the poly(A)-tail and could contain conserved, highly structured motifs like s2m, “barbell-like” structures, or sequence repeats like the “q-motif” [11,12,13]. 

According to the current Virus Taxonomy, the picornaviruses are classified into 147 species which belong to 63 genera [14]. All of the currently identified PVs are known to infect only vertebrate species where the infection-related disease spectrum could be ranged between asymptomatic to severe symptoms, including hepatitis (e.g., human hepatitis A, duck hepatitis A virus), encephalitis (e.g., EV-71, avian encephalomyelitis) or respiratory syndrome (e.g., human rhinovirus, bovine rhinitis A virus) [2,12,15]. Although, the in vitro cultivation of certain “classical” animal picornaviruses, like entero-, aphtho- or sapeloviruses are mostly possible in different, widely used cell lines (like MDBK or PK-15), even so, a number of recently identified PVs have failed to be cultured in vitro so far [16,17,18]. 

While the majority of the PVs were identified from mammal hosts, including domestic animals, like bovine (*Bos taurus*) or swine (*Sus scrofa*) (i.e., there are known bovine and swine PVs from a total of sixteen genera), only a limited number of PVs have been described from ovine (*Ovis aries*) and caprine (*Capra aegagrus hircus*) of similar economic importance [19]. Besides certain highly pathogenic PVs with wide host species spectra like foot-and-mouth disease virus (FMDV, *Aphthovirus*), or encephalomyocarditis virus (EMCV, *Cardiovirus*), only a few additional caprine (EV-F1, EV-G20) and ovine enteroviruses (EV-E1, G5, G7) of genus *Enterovirus*, a caprine (caprine kobuvirus 1) and ovine kobuvirus (ovine kobuvirus 1) of genus *Kobuvirus*, as well as an ovine hunnivirus (*Hunnivirus*) and an ovine boosepivirus (*Boosepivirus*), were discovered so far [20,21,22,23,24,25,26].

To date, only a single, currently uncharacterised (i.e., no related studies are available) genomic sequence of bovine picornavirus 1/bopivirus A1 strain TCH6 (KM589358) of genus *Bopivirus* identified from bovine in 2013 is available in the GenBank database without any supporting information. In this study, three novel PVs provisionally called ovipi-, gopi- and bopi-virus/Hun were identified from faecal samples of asymptomatic cloven-hoofed domestic livestock (ovine, caprine and bovine, respectively), which are most likely belonging to various genotypes of the existing species of *Bopivirus A* and a novel proposed species of “Bopivirus B” (introduced in this study) of genus *Bopivirus*. The presumably complete genomes of all three viruses were determined, characterised and an RT-PCR-based epidemiological investigation, as well as VP1 capsid sequence-based genotype-determination of bopiviruses, were also conducted using enteric samples of various species of domestic livestock (ovine, caprine, bovine, swine and rabbit). 

## 2. Materials and Methods 

### 2.1. Background Information of Samples, Animals and Farms

**Figure 1:**
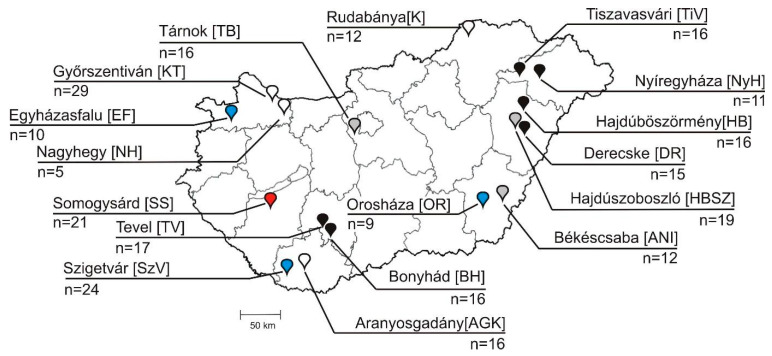
Localisations with farm IDs (in square brackets) and numbers of collected faecal samples (n = 269) of different Hungarian animal farms used for investigation of bopiviruses. Empty markers: caprine farms, black markers: bovine farms, grey markers: ovine farms, red marker: rabbit farm, blue markers: swine farms.

In this study, a total of 269 faecal samples were used, collected from mostly asymptomatic (only N = 11 caprine and N = 6 bovine were diarrheic) cloven-hoofed animals of *Ovis aries* (domestic ovine, N = 47), *Capra aegagrus hircus* (domestic caprine, N = 62), *Bos taurus* (domestic bovine, N = 96) and *Sus scrofa domestica* (domestic swine, N = 43), along with N = 21 samples of *Oryctolagus cuniculus domesticus* (domestic rabbit) between 2008 and 2020 (Table 1, Supplementary Table S1). A total of 17 distinct farms (N = 3 ovine, N = 4 caprine, N = 6 bovine, N = 3 swine and a single rabbit farm) were included in this study (Figure 1, Supplementary Table S1).

The majority of the investigated farms were “large-scale industrial livestock farms”, in which ≈100 to >1000 animals were housed together, except caprine farms of Nagyhegy (≈10 animals), Aranyosgadány (≈50 animals) and a bovine farm of Nyíregyháza (≈20 animals). All of the ovine and caprine (except for Győrszentiván) farms, as well as a single bovine farm (Derecske), were extensive-management-type farms where animals of different ages were released freely during the daytime and were housed together indoors at night. In contrast, the other N = 5 bovine, all swine, all rabbit and a caprine farm of Győrszentiván were intensive-management-type farms, where animals were restricted to a closed indoor farm area at all times. In the investigated intensive-management-type bovine farms, the <2-month-old calves were mostly held individually in separated areas. Faecal specimens were taken from the flooring underneath the animals into sterile test tubes, transported in chilled containers and kept at −20 °C prior to processing. For preliminary epidemiological investigations, the available sampled animals were retrospectively classified into three arbitrary age groups (group I: <2 months old, group II: 2–12 months old, group III: >12 months old) (Table 1, Supplementary Table S1). 

### 2.2. RT-PCR-Based Screening and Genome Acquisition Reactions

Total RNA was extracted from 150 µL of faecal sample diluted to ~40 *v/v*% with sterile 0.1 M phosphate-buffered saline (PBS) or 150 µL of cell-culture supernatant using TRI Reagent (MRC, Cincinnati, OH, USA) according to the manufacturer’s instructions. For complete genome determination, the RT-PCR technique was used, along with two types of 5′/3′ RACE (rapid amplification of cDNA ends) methods, terminal deoxynucleotidyl transferase (TdT) enzyme-based (Roche Diagnostics, Mannheim, Germany) [27], and adapter ligation methods using a T4 RNA ligase-based system [28]. The applied 5′ RACE protocols included the use of various reverse transcriptases (M-MLV-RT, Promega, USA; Maxima H-minus RT, Thermo-Fisher, Waltham, MA, USA) and the synthesis of either poly cytosine, guanine or adenine tracks with the use of TdT. Generic oligonucleotide primers used for the initial primer-walking-type genome acquisition of ovipivirus were designed based on the nucleotide (nt) alignments of bopivirus A1 (KM589358) and the most closely related sequences of erboviruses (genus *Erbovirus*). Further primers used for the genome acquisition of gopivirus and bopivirus/Hun were designed based on the nt alignment of bopivirus A1 (KM589358) and ovipivirus (data not shown). For the 3D^RdRp^-based screening and VP1 capsid-based typing RT-PCR reactions of bopiviruses, multiple sets of generic primer pairs were designed and used (Supplementary Table S2). 

The conditions and reagents used in the genome acquisition and screening/typing RT-PCR reactions were the same as described previously, with minor modifications [21]. Briefly, in the RT reactions, 1 μL of total RNA was applied in a final volume of 25 μL, and the total volume of the RT product was used in the PCR reaction in a final volume of 50 μL. The PCR thermal programs contained a total of 39 cycles. Details about the oligonucleotide primers used in the screening and typing RT-PCR reactions can be found in Supplementary Table S2. The generated PCR products were sequenced directly in both directions using a BigDye Terminator v1.1 Cycle Sequencing Kit (Thermo Fisher) on an ABI 3500 Genetic Analyzer (Applied Biosystems, Hitachi, Tokyo, Japan).

### 2.3. In Silico Sequence and Phylogenetic Analyses

Newly acquired sequences were searched against the GenBank database using the similarity search tools of BLASTn/x [29,30]. Multiple sequence alignments used for primer design, phylogenetic analyses and sequence comparisons were produced on the online platform of Multiple Sequence Comparison by Log-Expectation [31]. GeneDoc software ver. 2.7 was used for sequence assembly, as well as pairwise nucleotide (nt) and amino acid (aa) identity calculations. Pairwise differences (p-distances) of aligned bopiviruses VP1 nt sequences were calculated using the MEGA-X software ver. 10.2.1 and a frequency distribution histogram was created using Microsoft Excel [32,33]. Nt and derived aa sequence-based phylogenetic trees were constructed either by the use of IQ-TREE webserver (3CD tree) or the MEGA-X software ver. 10.2.1 (all of the other trees), with maximum likelihood method and various models specified in the legends of relevant figures [34]. Possible proteolytic cleavage sites of the study viruses were predicted based on the individual aa alignments with *Equine rhinitis B* viruses [35]. The potential secondary RNA structures of the 5′ UTRs and *cre* of the study viruses were created using the Mfold software [36]. For the visual representation of predicted RNA folding models, VARNA and CorelDraw Graphics Suite v. 12. software was used [37]. SimPlot software ver. 3.5.1 was applied for distance plot calculations using the Hamming model with a window size of 200 nt and a step size of 20 nt [38]. 

The complete genomic sequences of ovipivirus strain ovine/TB14/2010-HUN, gopivirus strain goat/AGK14/2020-HUN and bopivirus/Hun strain bovine/TV-9682/2019-HUN, the N = 27 partial 3D^RdRp^ and N = 16 partial VP1 sequences are all available in the GenBank database under the accession numbers MW298057–MW298059, MW298060–MW298086 and MW298087–MW298102, respectively.

### 2.4. Cell Culture and Virus Inoculation

Mardin-Darby bovine kidney (*Bos taurus*, MDBK), bovine lung (IPB3), Opossum kidney (*Didelphis virginiana*, OK) and swine kidney (*Sus scrofa*, PK-15) cell lines, as well as primary calf lung and testis cells, were cultured at 37 °C in a 5% CO_2_ humidified atmosphere in two different media. The MDBK, IPB3 and PK-15 cells were grown in Dulbecco’s Modified Eagle Medium:Nutrient Mixture F-12 (DMEM-F12, Gibco/Thermo Fisher, Waltham, MA, USA) supplemented with 10% foetal calf serum (FCS) and 10 mg/L gentamycin antibiotic solution (Sigma-Aldrich, St. Louis, MI, USA), while the other cells were incubated in Eagle’s Minimum Essential Medium (EMEM) supplemented with 2 mM of L-glutamine (Sigma-Aldrich, St. Louis, MI, USA) and 10% FCS. Faecal suspensions (≈40 *v*/*v*% in 0.1M PBS) used for inoculations were first centrifuged (10,000× rpm, 10 min) then the supernatants were passed through 0.45 µm sterile membrane syringe filters (Millipore, Bedford, MA, USA). The presence of bopivirus RNA in the filtrates was verified using bopivirus screening RT-PCR reactions. The filtered undiluted and the serial 10-fold diluted samples up to 10^−3^ dilutions were then inoculated on 80–90% confluent cells grown in either sterile 25 cm^2^ cell culture flasks or in 24-well plates. 500 µL of the prepared inoculums were incubated with each of the selected cell lines for 60 min at 37 °C, followed by addition of fresh medium. After 4 days of incubation, the cell cultures were assessed for cytopathic effects (CPE). Following repeated freeze-thawing and centrifugation (1000× *g* for 10 min), the culture supernatants were transferred to fresh cell cultures (passages). Up to six passages with similar steps were applied. After every passage, cells and supernatants were collected and archived at −80 °C for further analyses. Viral growth (presence of bopivirus RNA in the culture supernatants after the first passage) was monitored using screening RT-PCR reactions.

## 3. Results

### 3.1. Genome Characterisation of Ovine Picornavirus (Ovipivirus)

**Figure 2:**
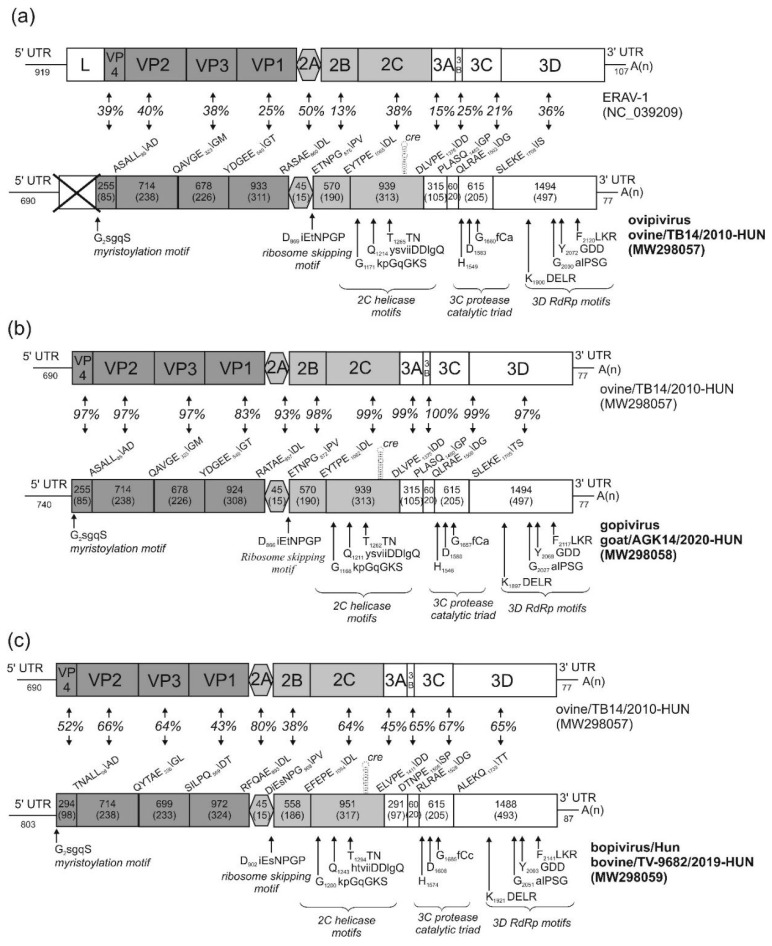
Schematic genome maps with conserved amino acid motifs and presumed cleavage sites (P5\P2′) of ovipivirus (**a**), gopivirus (**b**) and bopivirus/Hun (**c**) (bottom side of the panels) and their closest relatives (top side). The gene boxes corresponding to the P1 viral capsid proteins and P2 non-structural proteins are highlighted with different shades of grey. The nucleotide (upper numbers) and amino acid (lower numbers in brackets) lengths of the corresponding genomic regions of the study viruses are shown in each gene box. The positions and sequences of conserved amino acid motifs of 2C Helicase, 3C Protease and 3D^RdRp^ (RNA-dependent RNA polymerase) are shown under each map of the study viruses. Conserved and variable amino acids are represented by uppercase and lowercase letters, respectively. The pairwise amino acid identity values (%) of each genomic region are found between the genome maps. *cre*: cis-acting replication element.

The initial aim of the study was to detect the recently described *Boosepivirus C* (or related) picornaviruses [26], in enteric samples of various livestock in Hungary by RT-PCR using the in-house designed generic OvEncePV-Screen-R/F primer pair (Supplementary Table S2). However, instead of *Boosepivirus C*, serendipitously, an 873 nt-long non-boosepivirus sequence was identified from an ovine faecal sample (sample ID: TB14), which showed 50% nt and 43% aa identity to the capsid region of bovine picornavirus 1/bopivirus A1 (KM589358) of genus *Bopivirus* as the closest match identified by BLASTn/x searches. From this sequence, the complete 7385 nt-long RNA genome (excluding the polyA-tail) of strain ovine/TB14/2010-HUN (MW298057) was determined using primer-walking techniques and 3′/5′ RACE PCR methods and provisionally named ovipivirus (ovine picornavirus, analogue to the naming of bopivirus = bovine picornavirus) (Figure 2a). 

**Figure 3:**
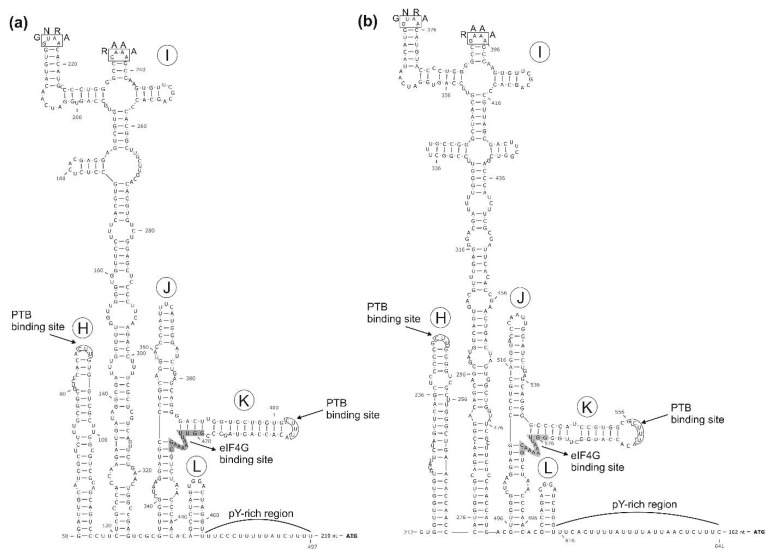
The predicted secondary RNA structures of 5′UTR-IRES core domains (H–L) of ovipivirus (**a**) and bopivirus/Hun (**b**). Numbers indicate the nucleotide positions in the complete genome of the given virus. PTB: Polypyrimidine tract-binding protein, eIF4G: Eukaryotic translation initiation factor 4 G, pY: polypyrimidine (C or U), **ATG**: Start codon.

The 5′ UTR is 690 nt-long, 440 nt longer than bopivirus A1 (KM589358) and shows only low (53%) pairwise sequence identity. Based on the results of secondary RNA structure modelling and analyses, the predicted IRES of the ovipivirus most likely belongs to type-II, similar as found in the related EMCV of genus *Cardiovirus* (Figure 3a). Besides the high structural resemblance between the core domains (domains H–L) of ovine/TB14/2010-HUN and EMCV IRESs, conserved sequence motifs (e.g., GNRA, RAAA) and transcription factor binding sites (e.g., eIGF4G, PTB) were also identifiable (Figure 3a) [39,40,41]. There is a c.a. 211 nt-long stretch with multiple presumed stem-loops (data not shown) between the poly(Y)-rich (Y = C or U bases) region of the IRES core domains and the presumed start codon of ovine/TB14/2010-HUN (Figure 3a). Moreover, although no conventional start codon is observable (only alternative start codons, e.g., C_492_UG are found) in this 210 nt-long stretch, it could be in silico translated into a 69 aa-long single protein with unknown function and with no significant sequence relationship identifiable by BLASTp searches. Furthermore, the length between the first domain (H) of the IRES and the 5′ terminal end is just 57 nt, therefore there is a possibility that the 5′ end is incomplete, although using different 5′ RACE approaches (i.e., heat-resistant reverse transcriptases, poly-(A), -(C) and -(G) tailing by TdT, adapter ligation), no additional sequences could be determined in any of the study sequences, including ovipivirus. The presumed *cre* with a single-stem-loop and a conserved AAAC-motif in the loop was observable at the 2C genome region between nt position 4314 and 4346 (Figure 2a, Supplementary Figure S1).

**Figure 4:**
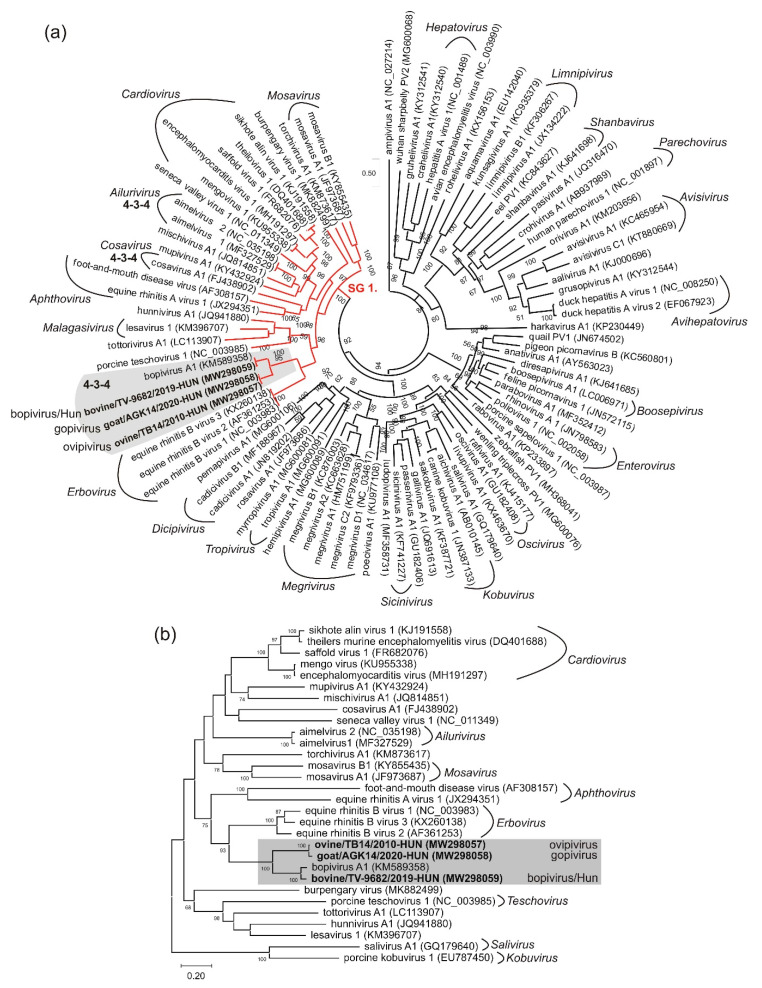
Phylogenetic analyses of the study ovipi-, gopi-, bopiviruses (in **bold**) and the representative members of family *Picornaviridae* based on the amino acid alignments of 3CD (**a**) and P1 (**b**) genomic regions. The trees are constructed using the Maximum likelihood method and either LG + F + I + G4 (a) or Jones Taylor Thornton (b) amino acid substitution models with 1000 iterations (bootstrap). The phylogenetic position of genus *Bopivirus* is marked with a grey background. The lineages of supergroup 1 (SG1) in the 3CD tree are marked with red lines. 4-3-4 indicate the genera in SG1 where picornaviruses with 4-3-4 genome organisations are present. The locations of some genera are shown in *italics* to help the orientation. A representative member of genera *Sali-* and *Kobu-virus* were used as outgroups in the P1 phylogenetic tree.

The start codon of the main ORF located in an optimal Kozak context (uuCACaA_691_UGG, start codon is underlined, conserved nts are capitalised). This 6618 nt-long ORF presumably encodes a 2205 aa-long single polyprotein which shows 57% nt and 58% aa sequence identity to the CDS of bopivirus A1 (KM589358), which is the closest match identifiable with BLAST searches. Since the single available sequence of bopivirus A1 is still vastly uncharacterised, for genome comparisons, the second most closely related sequence (based on BLAST analyses), equine rhinitis A virus 1 (ERAV-1, NC_039209), was used, which was previously analysed in detail [35]. The distribution of predicted cleavage sites suggests that the ovine/TB14/2010-HUN has a typical 4-3-4 genome organisation pattern where no N-terminal Leader peptide could be observed (Figure 2a). The aa sequence identities range between 13% (2B) and 50% (2A) of the study strain and ERAV-1 (Figure 2a). Based on the presence of conserved aa motifs, the N-terminal end of VP4 could be myristoylated and the single, short (15 aa-long) 2A peptide contains the “ribosome skipping” site of DxExNPG↓P (x = variable aa, conserved aa are capitalised), and therefore belong to the aphthovirus-like 2A-type [42,43]. The presumed functionally active sites of 2C^Hel^, 3C^Pro^ and 3D^RdRp^ are also present [44,45,46] (Figure 2a). The 3′ UTR of ovine/TB14/2010-HUN is 77 nt-long and contains no detectable sequence repeats or conserved motifs and shows only 48% nt identity to the 87 nt-long 3′UTR of bopivirus A1 (KM589358) (data not shown). Phylogenetically, the ovipivirus was clustered together with bopivirus A1 in the 3CD and P1 phylogenetic trees, and most likely belongs to the same genus, *Bopivirus* (Figure 4).

**Figure 5:**
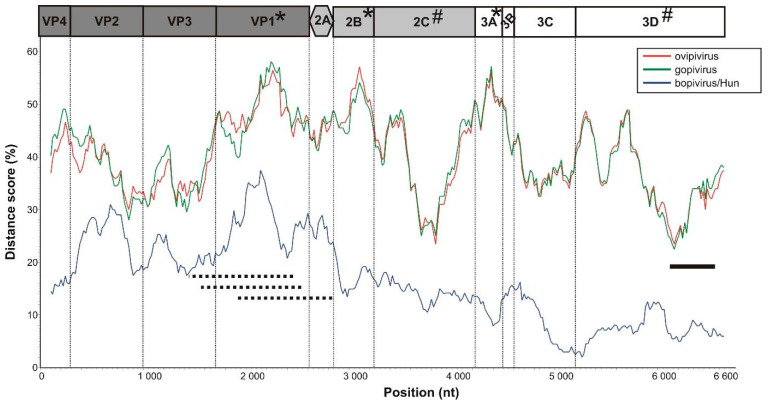
Distance plot of ovipi-, gopi-, and bopi-virus/Hun compared to the bopivirus A1 (KM589358) as a query sequence (reference) in a multiple nt alignment with a window size of 200 bp and a step size of 20 bp. Each line is a comparison between the CDS of ovipivirus (red), gopivirus (green) and bopivirus/Hun (blue) and the reference genome. Horizontal black line: the position of the RT-PCR product generated by the generic bopivirus screening oligonucleotide primers (HBG-3D-Screen-R/F). Horizontal dotted lines: positions of the RT-PCR products generated by the multiple primer sets used in various bopivirus typing RT-PCR reactions (Supplementary Table S2). Vertical dotted lines: borders of given genome regions indicated in the genome map above the plot. The most diverse (*) and most conserved (#) genome regions between ovipi-, gopi- and bopi-viruses are indicated in the bopivirus genome map.

Based on the distance plot analysis between the ovipivirus and bopivirus A1 sequences, the most diverse (VP1, 2B and 3A) and most conserved (2C^Hel^ and 3D^RdRp^) genome parts could be identified (Figure 5). Using these results, the generic HBG-3D-Screen-R/HBG-3D-Screen-F bopivirus screening primers were designed to the most conserved part of 3D^RdR^ of ovipi- and bopi-viruses (Supplementary Table S2).

### 3.2. Genome Characterisation of Caprine Picornavirus (Gopivirus) 

The complete genome of a novel ovipi/bopivirus-related picornavirus—provisionally called gopivirus (goat picornavirus)—was determined from a generic bopivirus screening RT-PCR-positive caprine faecal sample (sample ID: AGK-14) using the same approaches (primer-walking techniques and 5′/3′ RACE PCR method) as used for the genome determination of ovipivirus. The genome of gopivirus strain goat/AGK14/2020-HUN (MW298058) was 7426 nt, excluding the poly(A)-tail (Figure 2b). The 740 nt-long 5′ UTR of gopivirus contains a 50 nt-long extension in the 5′ end which was not detectable in the 5′ terminal end of ovipivirus. The 5′ UTR sequences of gopi- and ovipiviruses show relatively high (96%) pairwise nt sequence identity without the 5′ extension. The gopivirus has type II-like IRES and the same 210 nt-long start codon-less (only alternative start codons, e.g., C_542_UG are found) extension between the poly(Y)-rich region and the presumed start codon of the main ORF, as found in the ovipivirus (data not shown). The presumed *cre* was also observable at the 2C genome region, similar as found in the ovipivirus (Figure 2b, Supplementary Figure S1). The start codon of the main ORF is located in an optimal Kozak context (uCCACaA_741_UGG, start codon is underlined, conserved nts are capitalised). The 6609 nt-long main ORF presumably encodes a 2202 aa-long single polyprotein, which shows 92% nt and 96% aa sequence identity to the CDS of ovipivirus (Figure 2b). The aa sequence identities range between 83% (VP1) and 100% (3B) of the gopivirus and ovipivirus (Figure 2b). The predicted cleavage sites, the genome organisation layout (4-3-4), the conserved aa motifs and the aphthovirus-like 2A type are generally the same as found in the genome of ovipivirus (Figure 2b). The 3′ UTR of goat/AGK14/2020-HUN is only 77 nt-long and contains no detectable sequence repeats or conserved motifs and shows 98% nt identity to the 3′ UTR of ovipivirus (data not shown). Phylogenetically, the gopivirus was clustered together with the ovipivirus, and formed a distinct lineage related to bopivirus A1 in the 3CD and P1 phylogenetic trees (Figure 4) and most likely belongs to the genus *Bopivirus*. 

### 3.3. Genome Characterisation of Bovine Picornavirus (Bopivirus/Hun) 

An additional complete genome of an ovipi-/bopivirus-related picornavirus, provisionally called bopivirus/Hun (bovine picornavirus/Hungary) was determined from a generic bopivirus screening RT-PCR-positive bovine faecal sample (sample ID: TV-9682). The possible full-length sequence of bopivirus/Hun strain bovine/TV-9682/2019-HUN (MW298059) shows overall 82% nt (93% aa) identity to bopivirus A1 (KM589358) of genus *Bopivirus,* which is the closest match identified by BLAST searches, and only 57% nt (58% aa) identity to the ovipivirus in this study. The complete genome of bopivirus/Hun was 7571 nt, excluding the poly(A)-tail, and predicted to contain a single ORF (Figure 2c). The 803 nt-long 5′UTR is 555/103 nt longer than bopivirus A1 (KM589358)/ovipivirus and show only 64% and 52% pairwise sequence identities. Based on the results of secondary structure predictions and the observable structural similarities as well as the presence of conserved sequence motifs (e.g., GNRA, RAAA) and transcription factor binding sites (e.g., eIGF4G, PTB), the bopivirus/Hun has a similar type II-like IRES as the ovipi- and gopi-viruses (Figure 3b). The stretch between the poly(Y)-rich region and the start codon was shorter (only 162 nt) compared to ovipi/gopiviruses and contains multiple stop codons in all three reading frames (data not shown). The presumed *cre* could also be present at the 2C genome region, similar as found in the ovipi- and gopi-virus (Figure 2c, Supplementary Figure S1). The start codon of the ORF is located in an optimal Kozak context (uuCACaA_804_UGG, start codon is underlined, conserved nts are capitalised). The 6681 nt-long ORF presumably encodes a 2226 aa-long viral polyprotein which shows 83%/57% nt and 93%/58% aa sequence identity to the CDS of bopivirus A1 (KM589358) and ovipivirus. The pairwise aa sequence identities of corresponding genomic regions of the bopivirus/Hun and ovipivirus ranged between 38% (2B) and 80% (2A) (Figure 2c). The bopivirus/Hun shows similar genome architecture (4-3-4), similar NPGP-containing “ribosome-skipping” type 2A and similar conserved functional sites as found in the ovipi- and gopi-viruses (Figure 2c). The 3′ UTR of bopivirus/Hun is 87 nt-long and contains no detectable sequence repeats or conserved motifs and shows only 36% nt identity to the 3′UTR of ovipi- and gopi-viruses but 97% nt identity to the 3′ UTR of bopivirus A1 (data not shown). Phylogenetically, the bopivirus/Hun is closely related to bopivirus A1, and located on the same lineage as ovipi- and gopi-virus in the 3CD and P1 phylogenetic trees (Figure 4), and therefore, most likely belongs to the genus *Bopivirus*. 

### 3.4. Epidemiological Investigation of Different Bopiviruses in Hungarian Animal Farms

In order to investigate the prevalence of different bopiviruses among livestock originated from geographically distant locations in Hungary, a total of N = 47 ovine, N = 62 caprine, N = 96 bovine, N = 43 swine and N = 21 rabbit faecal samples (Figure 1, Table 1, Supplementary Table S1) were screened by RT-PCR using HBG-3D-Screen-R/HBG-3D-Screen-F primer pairs (Supplementary Table S2). The overall RT-PCR positivity by host species was 36.2%, 25.8% and 4.2% in the analysed ovine, caprine and bovine populations. None of the analysed swine and rabbit faecal samples were found to be RT-PCR-positive (Table 1, Supplementary Table S1). 

Among the investigated farms, the RT-PCR positivity was up to 62.5% (10/16 positive, Tárnok) in ovine farms, 50.0% (8/16 positive, Aranyosgadány) in caprine farms and 11.8% (2/17 positive, Tevel) in bovine farms (Table 1, Figure 1). Positive animal farms are found in different regions of Hungary (Figure 1, Table 1, Supplementary Table S1). From the total of N = 13 different animal farms examined, bopivirus was detectable in N = 8 geographically distant sites (Table 1, Figure 1). 

**Figure 6:**
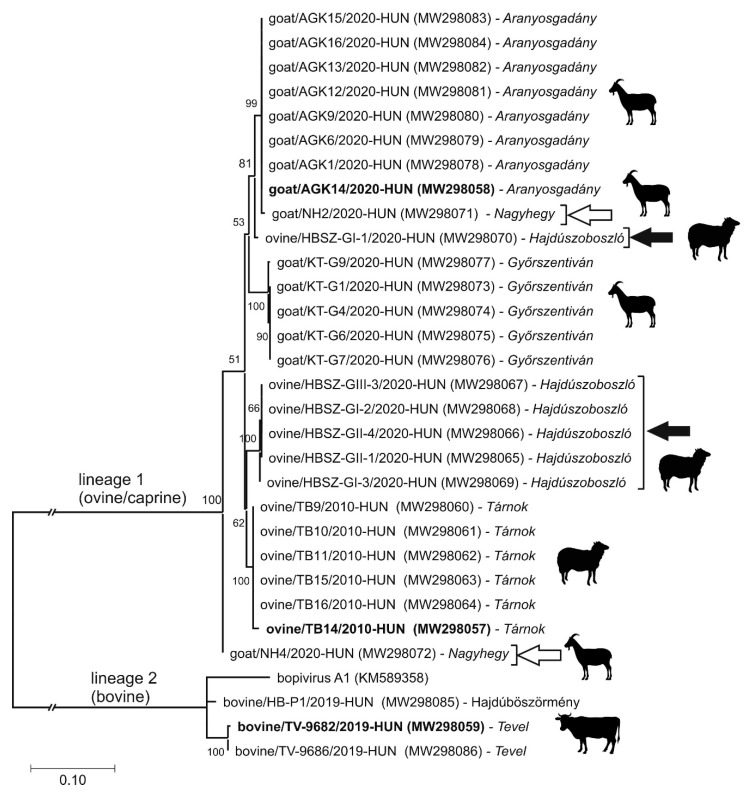
Phylogenetic analysis of determined bopivirus 3D^RdRp^ sequences. The tree was constructed with the Maximum Likelihood method and Jukes-Cantor model. The percentage of trees in which the associated taxa clustered together (bootstrap) is shown (where values are >50) next to the branches. The trees are drawn to scale, with branch lengths measured in the number of substitutions per site. The origin (farm location) and silhouettes of the samples’ hosts are found after the strain names. The locations of lineage 1 of ovine/caprine and lineage 2 of bovine bopiviruses are also indicated. Black and empty arrows indicate the phylogenetic positions of different caprine (empty arrows) and ovine (black arrows) lineages originated from the same farm. The ovipi-, gopi- and bopi-virus/Hun sequences are marked in **bold**.

From the total of N = 269 analysed samples, only N = 17 (11 caprine and 6 bovine) were collected from symptomatic (gastroenteritic, GE) animals, and only two of these samples were positive for bopivirus by RT-PCR screening (Supplementary Table S1). Other co-infecting pathogens which could cause GE were not investigated. Based on the results of pairwise sequence comparisons and phylogenetic analysis of the 587 nt-long partial 3D^RdRp^ sequences determined by Sanger-sequencing from the screening RT-PCR reactions, two main bopivirus groups could be distinguished (Figure 6). Sequences of the first group from ovine and caprine hosts share 95.4–100% nt identities, while sequences from the second group (bovine) show 91.7–99.7% pairwise identity values (data not shown). The nt sequence identities are considerably lower between the two groups: sequences from ovine and bovine share only 69–70% nt identities, while sequences of caprine and bovine also show low values (69.3–70.4%) (data not shown). The two bopivirus sequence groups (ovine/caprine and bovine sequences) are also clearly separated in the 3D^RdRp^ phylogenetic tree and form two distinct, monophyletic lineages (Figure 6). 

The sequences from ovine and caprine share similarly high identities and are located on the same mixed ovine/caprine lineage, and therefore could not be distinguished from each other (Figure 6). The 3D^RdRp^ sequences from the first group/lineage (ovine and caprine) show the highest sequence identities (95.9–100%) and cluster together with the corresponding genome parts of ovipi- and gopi-viruses, while sequences from bovine are most closely related (91.7–99.7%) to the bopivirus/Hun and bopivirus A1 (Figure 6). Sequences from the same farm are usually clustered together, although there are farms like Nagyhegy (caprine) and Hajdúszoboszló (ovine) where multiple, phylogenetically distant variants are detectable (Figure 1, Figure 6). While, bopiviruses were found to be the most prevalent among the <2-month-old ovine (group I, 66.7%) followed by the 2–12-month-old group (II, 60%), and no RT-PCR positivity was found in adult (>12-month-old) animals (group III). Until then, these viruses were not or just rarely present among young (<2-month-old) caprine (0%) and bovine (2.2%), and most frequently detectable among older, group II (40%, 6.5%) and group III (17.4%, 5.6%) animals examined (Table 1). 

### 3.5. VP1-Capsid-Based Analyses of Different Bopiviruses

In order to investigate the more detailed genetic/genotypic variance of the different bopivirus field strains, based on the results of distance plot (Figure 5), the most diverse (VP1-capsid encoding) genome region was chosen for target of RT-PCR-based acquisition reactions and further in silico analyses.

**Figure 7:**
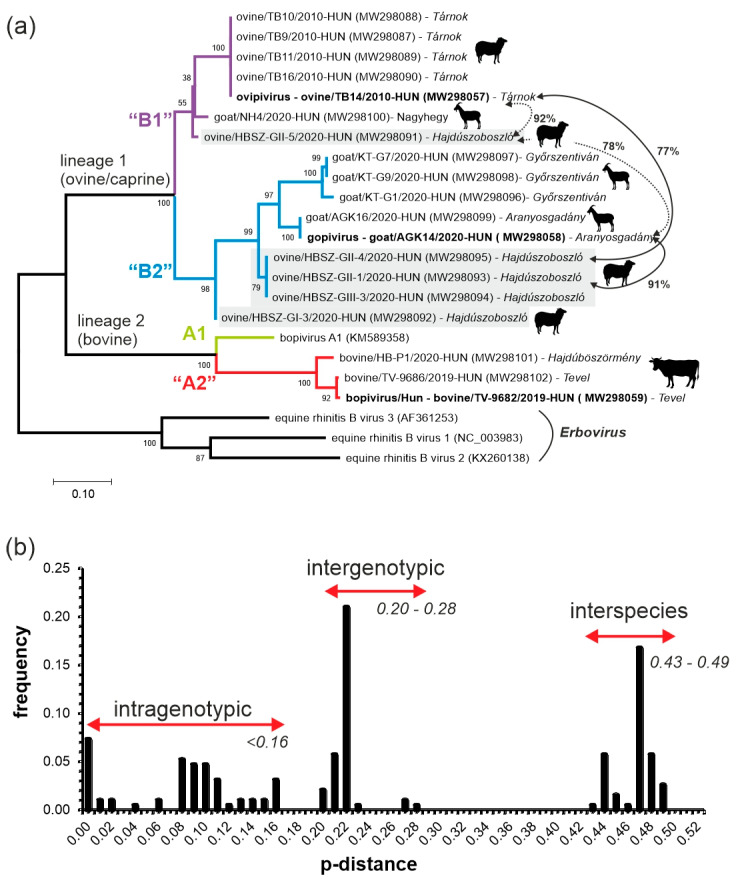
(**a**) Phylogenetic analysis of the determined bopivirus VP1 sequences with three different genotypes of genus *Erbovirus* which was used as an outgroup. The tree was constructed using the Maximum Likelihood method and Kimura 2 model. The percentage of trees in which the associated taxa clustered together (bootstrap) is shown (if its values are >50) next to the branches. The trees are drawn to scale, with branch lengths measured in the number of substitutions per site. The origin (farm location) and silhouettes of the samples’ hosts are found after the strain names. The locations of lineage 1 of ovine/caprine and lineage 2 of bovine bopiviruses are also indicated. The diverse ovine strains from Hajdúszoboszló are highlighted with a grey background. Percentages are pairwise nt identity values between strains indicated by different double arrows. The ovipi-, gopi- and bopi-virus/Hun sequences are marked with **bold**. The branches of the strains belonging to different existing (A1) and proposed genotypes (“A2”, “B1”, “B2”) are marked with different colours. (**b**) Frequency distribution of pairwise distances (p-distances) between the N = 20 VP1 nucleotide sequences of all study strains and bopivirus A1 reference sequence with intra-, inter-genotypic and possible interspecies distance ranges in *italics*.

From the total of N = 37 bopivirus screening RT-PCR-positive faecal samples collected from 8 different farms, only N = 16 VP1 sequences from seven farms could be determined (excluding the strains with determined complete genomes) using multiple oligonucleotide primer sets (Supplementary Tables S1 and S2, Figure 7a). The acquired 813–931 nt-long VP1 sequences show 41.8–100% nt and 34–99% aa pairwise sequence identities to each other. VP1 sequences from the same host show 77.7–100% (ovine-origin), 77.1–99% (caprine-origin) and 87.6–93.5% (bovine-origin) pairwise nt identities (data not shown). The phylogenetic analysis of the determined VP1 sequences show the separation of the mixed ovine-/caprine-origin and bovine-origin bopiviruses into two distinct monophyletic lineages (lineages 1 and 2) (Figure 7a). Furthermore, there is a clear separation of further clusters within the lineages. The two clusters in lineage 1 could represent two different genotypes (“B1” and “B2”), and both contain mixed ovine and caprine bopivirus strains, while in lineage 2, the closely related Hungarian bopiviruses of bovine could also belong to a different genotype (“A2”) which is clearly separated from the reference sequence of Bopivirus genotype A1 (Figure 7a). Three separate regions could be identified in the frequency distribution histogram of pairwise distance (p-distance) scores among bopivirus VP1 sequences, which could correspond to the scores of intragenotypic (less than 0.16, mean 0.08), intergenotypic (between 0.20 and 0.28, mean 0.24) and possible interspecies (between 0.43 and 0.49, mean 0.46) variations (Figure 7b). The closely related VP1 sequences from animals of the same host/farm generally clustered together, except for sequences from Hajdúszoboszló (HBSZ) where multiple diverse lineages/genotypes could be observed (Figure 7a). The ovine/HBSZ-GII-4/2020-Hun, ovine/HBSZ-GII-1/2020-Hun and ovine/HBSZ-GIII-3/2020-Hun strains from Hajdúszoboszló are located among caprine-origin gopivirus sequences and show much higher sequence identity (91%) to gopivirus than to ovipivirus (77%), while the ovine/HBSZ-GII-5/2020-Hun strain from the same farm shows much closer phylogenetic (Figure 7a) and sequence relationship (92% nt identity) to ovine ovipiviruses than caprine gopiviruses (78 % nt identity). Furthermore, the goat/NH4/2020-Hun strain from farm Nagyhegy is separated from the other caprine-origin sequences from Aranyosgadány and Győrszentiván but clustered together with ovine bopiviruses (Figure 7a).

### 3.6. Results of Virus Cultivation 

In addition to the faecal samples of ovipivirus strain ovine/TB14/2010-HUN (TB14), gopivirus strain goat/AGK14/2020-HUN (AGK14) and bopivirus/Hun strain bovine/TV-9682/2019-HUN (TV-9682), two additional bopivirus screening RT-PCR-positive faecal samples of ovine (HBSZ-GII-5) and bovine (TV-9686) were randomly selected for cultivation attempts using six different swine (PK-15), opossum (OK) and bovine (MDBK, IPB3, primary testis and lung) cell lines. Visible cytopathic effect (CPE) was observed only in the bovine MDBK cells inoculated with bovine faecal samples of TV-9686 and TV-9682 after the first passage (data not shown). None of the other cell cultures showed any signs of CPE even after up to six subsequent passages. The bopivirus screening RT-PCR reactions showed no visible products in the expected size in any of the culture supernatants, including the ones with visible CPE.

## 4. Discussion

In this study, three novel picornaviruses, ovipivirus, gopivirus and bopivirus/Hun from faecal samples of cloven-hoofed domestic livestock (ovine, caprine and bovine) in Hungary were identified and genetically characterised using RT-PCR and dye-terminator sequencing methods. These three monophyletic viruses share the same type II-like IRES, aphthovirus-like ‘ribosomal skipping’-type 2A, similar genome layout (4-3-4) and *cre*-localisations. Furthermore, these viruses show the closest sequence and phylogenetic relationship to a currently uncharacterised reference sequence of bopivirus A1 (KM589358) of genus *Bopivirus*. Based on the overall high sequence identities (93% aa) and close phylogenetic relationship with bopivirus A1 in the complete P1 and 3CD phylogenetic trees, the bovine bopivirus/Hun strain bovine/TV-9682/2019-HUN most likely belongs to the *Bopivirus A* species, but based on the calculated p-distance value of VP1 capsid (0.27, which is between the intergenotypic p-distance range of 0.20–0.28) and the position in VP1 phylogenetic tree, it could be a distinct genotype (“bopivirus A2”). Although the ovipivirus strain ovine/TB14/2010-HUN is phylogenetically distinct from the bopivirus A1 and bopivirus/Hun in the P1, 3CD and VP1 trees, the level of aa sequence divergence between bopivirus/Hun and ovipivirus in the P1 (45%), 2C (36%) and 3CD (33%) genomic regions do not meet the classification criteria of being the founding member of a novel genus but suggest that ovipivirus could belong to a novel species (“Bopivirus B”) within the genus *Bopivirus* [14]. This classification was also supported by the calculated p-distance value between the VP1 of ovipivirus and bopivirus/Hun (0.47), which is in the range of calculated interspecies p-distance values (0.43–0.49). Interestingly, the gopivirus strain goat/AGK14/2020-HUN identified from a caprine shows significant sequence identity (overall 96% aa identity) and close phylogenetic relationship in the P1 and 3CD phylogenetic trees to the ovipivirus of ovine. Therefore, gopivirus strain goat/AGK14/2020-HUN could belong to the same species “Bopivirus B”, although based on the relatively low sequence identity (83%) in the VP1 capsid region, and the p-distance values of VP1 (0.22 which is between the intergenotypic p-distance range of 0.20–0.28), it could belong to a second genotype “bopivirus B2”. Some other examples are known of the close phylogenetic/sequence relationship observed between PVs of different cloven-hoofed animals, e.g., closely related viruses of species *Enterovirus F* and *G* of genus *Enterovirus*, as well as *Aichivirus B* of genus *Kobuvirus,* are also identified from bovine, ovine and caprine hosts [20,25,47]. 

In contrast to the majority of the genera (12 of the 15) of supergroup I, bopiviruses have no identifiable N-terminal Leader protein at the 5′ end of the CDS. Besides bopiviruses, only the members of genus *Ailurivirus* and *Cosavirus* are lacking the Leader protein and have 4-3-4 genome layout [2,14] (Figure 4). Interestingly, while the conserved poly(Y)-rich region adjacent to the core domains of type-II IRES is usually located only c.a. 9–17 nt upstream of the start codon [48], in the case of the ovipi- and gopi-virus, there is a c.a. 211 nt-long stretch between the poly(Y)-rich region and the presumed start codon of the viral polyprotein (Figure 3a). This stretch could be in silico translated (only alternative start codons are present) into a protein sequence with unknown function, and no significant sequence hit by BLAST searches. This region could be the remnant of the once active Leader encoding genome region of bopiviruses with increased mutation rate, which is supported by the presence of multiple stop codons and low sequence identity (30%) between the stretch of bopivirus/Hun and bopivirus A1 compared to the adjacent sequences of IRES core (80% nt identity). On the other hand, the presence of multiple predicted stem-loops in the 5′UTR stretches could indicate the similar structural role of this region as the domain A–G in type-II IRESs [48]. Furthermore, a similarly long (≈250 nt) nucleotide stretch between the poly(Y)-rich region and presumed start codon was also found in the Leader-containing avian siciniviruses with type-II IRES [49]. Experiments on cultured viruses should be conducted to address these hypotheses. Unfortunately, any attempts, including the use of multiple (up to six) passages for the cultivation of the study bopiviruses in six different bovine, opossum and swine cells were all unsuccessful. It is currently not known whether the inoculated bopiviruses were unable to grow in the selected cell lines, or the samples did not contain replication-competent viruses. 

The preliminary epidemiological investigation of different bopiviruses on a relatively low number of available faecal samples (N = 269) was based on the use of an “in-house designed” generic bopivirus screening primer pair (HBG-3D-Screen-R/F) in RT-PCR reactions and dye-terminator sequencing. The screening primers, which are targeting the most conserved genome region (3D^RdRp^) of different bopiviruses identified by distance plot analyses (Figure 5), were designed for the specific amplification of all of the currently known bopiviruses. The results of phylogenetic and sequence comparisons of partial 3D^RdRp^ sequences determined by screening RT-PCR reactions show a close relationship of the sequences of ovine/caprine (group 1) with the study ovipi- and gopi-virus as well as the sequences of bovine (group 2) with bopivirus/Hun and bopivirus A1 (Figure 6). Therefore, the identified ovine/caprine sequences of group 1 most likely belong to the candidate species “Bopivirus B”, while the group 2 sequences of bovine are part of the species *Bopivirus A*. The discrimination of caprine-origin and ovine-origin bopiviruses is not possible based solely on the analyses of partial 3D^RdRp^ sequences of screening RT-PCR reactions. No other viral sequence unrelated to bopiviruses was identified, suggesting the high specificity of the primers to bopiviruses which therefore could be used in diagnostic RT-PCR reactions as well as—by sequencing its products—the discrimination of *Bopivirus A* and “Bopivirus B” viruses. 

The presence of bopiviruses was predominantly investigated among asymptomatic animals, only a low number of samples (N = 17) from symptomatic (i.e., gastroenteritic) livestock were available, and only two of them were bopivirus RT-PCR-positive; therefore, due to the low number of samples from diseased animals, any role of bopivirus infection in the development of enteric (or any other) symptoms or extraintestinal infections are not investigated, and thus cannot be ruled out. There are other PVs like sapeloviruses or polioviruses which are capable of causing mostly asymptomatic infections, but in certain cases, it could cause diseases with sometimes serious (e.g., CNS involvements) manifested symptoms [50,51]. Bopiviruses could not be detected in the investigated rabbit (N = 21) and swine (N = 43) enteric samples using generic bopivirus screening RT-PCR reactions, which could indicate the absence of genetically related virus(es) detectable by the screening primers in the analysed samples. 

Although, there was a discrepancy in the prevalence of bopiviruses among age groups of different hosts, which could come from the uneven and in some cases a low number of sampled animals of different ages (i.e., ovine GII and caprine GI groups, Table 1), but based on these preliminary epidemiological results, bopivirus infection is likely to be more frequent among young, <12-month-old animals (age groups I, II) than in adults (group III, Table 1), similar as found in other picornaviruses as well [52,53,54]. Interestingly, despite the higher number of available samples of bovine (N = 96) compared to ovine (N = 47) and caprine (N = 62), the prevalence of bopiviruses among investigated bovine are still much lower (4.2%) than the prevalence data identified in ovine (36.2%) and caprine (25.8%). This could be due to the different housing conditions of <2-month-old calves, which were mostly held in individually separated areas in the investigated intensive-management farms compared to ovine and caprine which were held in herds together with older animals. In this case, viruses of older animals could serve as initial sources of bopivirus infection of calves. The presence of different bopiviruses in geographically distant animal farms, both in recently collected (in 2019 and 2020) and archived (in 2008 and 2009) samples, suggest the endemic presence and continuous, long-standing circulation of these viruses in the investigated domestic animal populations in Hungary.

Besides the use of different sets of universal primers targeting various parts of the P1 and P2 genome regions (Supplementary Table S1) in multiple RT-PCR reactions, VP1 sequences could only be determined from less than half (N = 16/37) of the bopivirus 3D^RdRp^ RT-PCR-positive faecal samples, which could indicate a high viral RNA sequence divergence of field strains of bopiviruses. This could be supported by the results of sequence comparisons (Figure 2) and the distance plot (Figure 5), where VP1 was found to be one of the most diverse regions of bopiviruses. Among the majority of PVs including, e.g., entero-, sapelo-, cardio- or the bopi-virus sister-clade of erboviruses, the VP1 capsid is the most diverse, and also the immunodominant viral peptide, which is responsible for the serotyping/genotyping of the viruses [8,9,10]. The phylogenetic analysis and the histogram of p-distances of determined bopivirus VP1 sequences supports the separation of ovine/caprine-origin and bovine-origin bopiviruses into two different species (*Bopivirus A* and “Bopivirus B”) as well as supports the classification of closely related lineages into different genotypes of bopivirus A1/”A2” and “bopivirus B1/B2”, and also indicates the endemic circulation of one or few, mainly farm-specific bopivirus geno/subtype(s) (Figure 7a,b). However, the calculated p-distance score ranges of intra-, inter-genotypic and interspecies variations should be re-evaluated when further bopivirus VP1 sequences will be available. Interestingly, while there are separate, caprine-origin gopivirus and ovine-origin ovipivirus lineages identifiable in the VP1 tree, there are a few ovine strains which are clearly located in the gopivirus lineage, belonging to the same genotype and also showing high sequence identity (91% nt) to gopivirus (Figure 7a), suggesting the possibility of ovine–caprine interspecies transmission of certain “Bopivirus B” strains, similar as suspected among certain ovine and caprine enteroviruses of species *Enterovirus G* [25]. 

Based on our results, the “neglected” (i.e., related publication not available since 2018) genus *Bopivirus* is most likely a species-rich group among picornaviruses which have high genotypic diversity, widespread geographical distribution (present in diverse regions of Hungary and the USA as well), wide host species spectra and currently unknown pathogenic potential. More detailed analyses including follow-up studies with extra-intestinal samples as well as large-scale epidemiological investigations of livestock are necessary to explore the true prevalence, pathogenesis, host spectra and genomic diversity of these novel picornaviruses.

## Figures and Tables

**Table 1:** Origin and features of sampled animals (hosts) as well as statistic data of bopivirus prevalence (in **bold**). Positive faecal sample refers to bopivirus screening RT-PCR positivity. (I), (II), (III): age groups. mo: month.

Host Species	Farm Location	Farm ID	Collection Date	No. of Positive Faecal Samples/Total by Age Groups			No. of Positive Faecal Samples/Total (%) by Farms
				(I) <2 mo	(II) 2–12 mo	(III) >12 mo	
Ovine	Hajdúszoboszló	HBSZ	05/03/2020	4/8	3/5	0/6	**7/19 (36.8%)**
	Tárnok	TB	02/04/2010	10/16	0	0	**10/16 (62.5%)**
	Békéscsaba	ANI	06/09/2009	0	0	0/12	**0/12**
Ʃ =				**14/21 (66.7%)**	**3/5 (60.0%)**	**0/18**	**17/47 (36.2%)**
Caprine	Aranyosgadány	AGK	23/04/2020	0	6/8	2/8	**8/16 (50.0%)**
	Győrszentiván	KT	11/05/2020	0/9	5/10	0/10	**5/29 (17.2%)**
	Nagyhegy	NH	11/05/2020	0	0	2/5	**2/5 (40.0%)**
	Rudabánya	K	25/06/2008	0	1/12	0	**1/12 (8.3%)**
Ʃ =				**0/9**	**12/30 (40.0%)**	**4/23 (17.4%)**	**16/62 (25.8%)**
Bovine	Hajdúböszörmény	HB	05/03/2020	1/6	0/13	1/2	**2/21 (9.5%)**
	Nyíregyháza	NyH	06/03/2020	0/7	0	0/4	**0/11**
	Derecske	DR	05/03/2020	0	0/4	0/11	**0/15**
	Tiszavasvári	TiV	05/03/2020	0/6	0/9	0/1	**0/16**
	Bonyhád	BH	04/11/2019	0/14	0/2	0	**0/16**
	Tevel	TV	04/11/2019	0/13	2/3	0	**2/17 (11.8%)**
Ʃ =				**1/46 (2.2%)**	**2/31 (6.5%)**	**1/18 (5.6%)**	**4/96 (4.2%)**
Swine	Egyházasfalu	EF	17/08/2016	0	0/10	0	**0/10**
	Orosháza	OR	15/09/2016	0/9	0	0	**0/9**
	Szigetvár	SzV	18/10/2018	0/24	0	0	**0/24**
Ʃ =				**0/33**	**0/10**	**0**	**0/43**
Rabbit	Somogysárd	SS	03/11/2010	0/13	0/4	0/4	**0/21**
Ʃ =				**0/13**	**0/4**	**0/4**	**0/21**

## Data Availability

Data available in a publicly accessible repository: The data presented in this study are openly available in GenBank database under the accession numbers MW298057–MW298102.

## Supplementary Materials

The following are available online at https://www.mdpi.com/1999-4915/13/1/66/s1, Figure S1: The predicted secondary RNA structure of presumed cis-acting replication elements, Table S1: Detailed background information of individual samples used for the epidemiological investigations of bopiviruses, Table S2: List of oligonucleotide primers used for screening and typing reactions of this study.
