# Supplementary material for: Multiple Types of Novel Enteric Bopiviruses (Picornaviridae) with the Possibility of Interspecies Transmission Identified from Cloven-Hoofed Domestic Livestock (Ovine, Caprine and Bovine) in Hungary
Source: Viruses. 2021 Jan 6;13(1):66. doi: 10.3390/v13010066 (PMC7825084; doi:10.3390/v13010066)
Supplement: Supplementary file 1 [file viruses-13-00066-s001.pdf]

viruses

Supplementary Materials - Multiple types of novel enteric bopiviruses (Picornaviridae) with the possibility of interspecies transmission identified from cloven-hoofed domestic livestock (ovine, goat and cattle) in Hungary

Zoltán László[1], Péter Pankovics[1], Gábor Reuter[1], Attila Cságola[2], Ádám Bálint[3], Mihály Albert[2], Ákos Boros[1]*

[1] Department of Medical Microbiology and Immunology, Medical School, University of Pécs, Pécs, Hungary; ifj.laszlozoltan@gmail.com; pankovics.peter@pte.hu; gabor.reuter@pte.hu, borosakos@gmail.com,
[2] Ceva Phylaxia Ltd. Budapest, Hungary; attila.csagola@ceva.com, mihaly.albert@ceva.com,
[3] Department of Virology, National Food Chain Safety Office Veterinary Diagnostic Directorate, Budapest, Hungary.; BalintAd@nebih.gov.hu,
* Correspondence: borosakos@gmail.com ; Tel.: +36 72 536-251

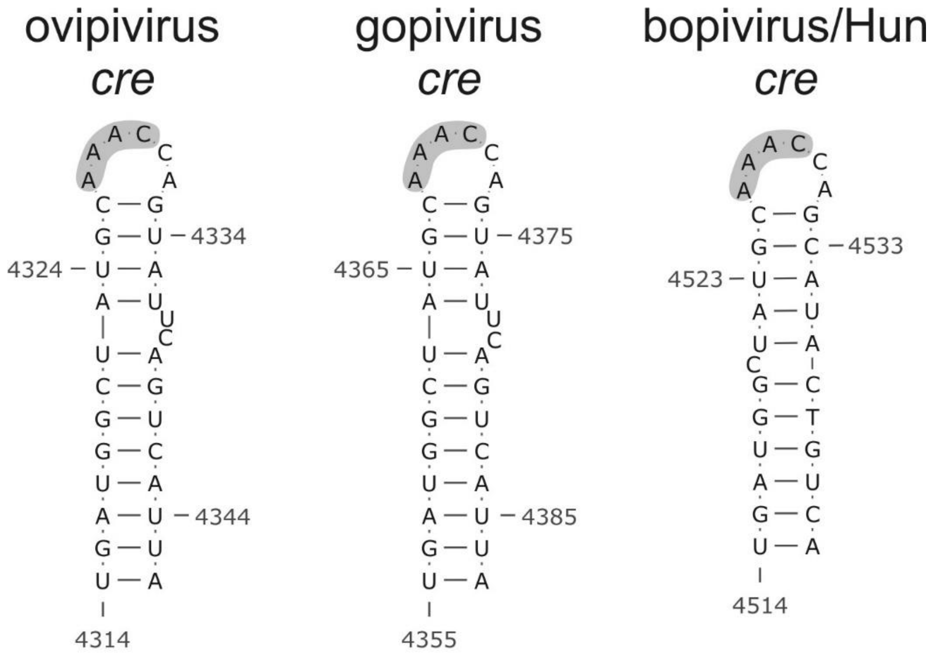

Figure S1.: The predicted secondary RNA structure of presumed cis-acting replication elements (*cre*) of ovipi-, gopi-, and bopivirus/Hun located in the $2C^{Hel}$ region. Numbers indicate the nt positions in the complete genome. Grey highlighted nts marked the location of conserved AAAC motif in the loop.

Table S1: Detailed background information of individual samples used for the epidemiological investigations of bopiviruses as well as the results of Bopi-3D^{RdRp} screening and Bopi-VP1 RT-PCR reactions. ID: identification marks. Age group I: < 2 month-old animals, Age group II. 2-12-month-old animals, Age group III: >12 month-old animals. n.t.: not tested (only when the prior Bopi-3D^{RdRp} screening was negative). The samples which were used for complete genome determination reactions are marked with an asterisk. AS: asymptomatic, GE: gastroenteritic animals. Bopi-3D^{RdRp}-screen RT-PCR positive samples are marked with **bold**.

Farm location (ID)	Sample ID	Host	Age group	Health status	Bopi-3D^{RdRp}-screen RT-PCR	Ovipi/Bopi-VP1 RT-PCR
Hajdúszoboszló (HBSZ)	**HBSZ-GI-1**	ovine	II	AS	+	-
Hajdúszoboszló (HBSZ)	**HBSZ-GI-2**	ovine	II	AS	+	-
Hajdúszoboszló (HBSZ)	**HBSZ-GI-3**	ovine	II	AS	+	+
Hajdúszoboszló (HBSZ)	HBSZ-GI-4	ovine	II	AS	-	n.t.
Hajdúszoboszló (HBSZ)	HBSZ-GI-5	ovine	II	AS	-	n.t.
Hajdúszoboszló (HBSZ)	**HBSZ-GII-1**	ovine	I	AS	+	+
Hajdúszoboszló (HBSZ)	HBSZ-GII-2	ovine	I	AS	-	n.t.
Hajdúszoboszló (HBSZ)	HBSZ-GII-3	ovine	I	AS	-	n.t.
Hajdúszoboszló (HBSZ)	**HBSZ-GII-4**	ovine	I	AS	+	+
Hajdúszoboszló (HBSZ)	**HBSZ-GII-5**	ovine	I	AS	+	+
Hajdúszoboszló (HBSZ)	HBSZ-GIII-1	ovine	I	AS	-	n.t.
Hajdúszoboszló (HBSZ)	HBSZ-GIII-2	ovine	I	AS	-	n.t.
Hajdúszoboszló (HBSZ)	**HBSZ-GIII-3**	ovine	I	AS	+	+
Hajdúszoboszló (HBSZ)	HBSZ-GIV-1	ovine	III	AS	-	n.t.
Hajdúszoboszló (HBSZ)	HBSZ-GIV-2	ovine	III	AS	-	n.t.
Hajdúszoboszló (HBSZ)	HBSZ-GV-1	ovine	III	AS	-	n.t.
Hajdúszoboszló (HBSZ)	HBSZ-GV-2	ovine	III	AS	-	n.t.
Hajdúszoboszló (HBSZ)	HBSZ-K-1	ovine	III	AS	-	n.t.
Hajdúszoboszló (HBSZ)	HBSZ-TL-1	ovine-pool	-	-	-	n.t.
Tárnok (TB)	**TB-1**	ovine	I	AS	+	-
Tárnok (TB)	TB-2	ovine	I	AS	-	n.t.
Tárnok (TB)	TB-3	ovine	I	AS	-	n.t.
Tárnok (TB)	**TB-4**	ovine	I	AS	+	-
Tárnok (TB)	**TB-5**	ovine	I	AS	+	-
Tárnok (TB)	TB-6	ovine	I	AS	-	n.t.
Tárnok (TB)	TB-7	ovine	I	AS	-	n.t.
Tárnok (TB)	**TB-8**	ovine	I	AS	+	-
Tárnok (TB)	**TB-9**	ovine	I	AS	+	+
Tárnok (TB)	**TB-10**	ovine	I	AS	+	+
Tárnok (TB)	**TB-11**	ovine	I	AS	+	+

Tárnok (TB)	TB-12	ovine	I	AS	-	n.t.
Tárnok (TB)	TB-13	ovine	I	AS	-	n.t.
Tárnok (TB)	**TB-14***	ovine	I	AS	+	+
Tárnok (TB)	**TB-15**	ovine	I	AS	+	-
Tárnok (TB)	**TB-16**	ovine	I	AS	+	+
Békéscsaba (ANI)	ANI-1	ovine	III	AS	-	n.t.
Békéscsaba (ANI)	ANI-2	ovine	III	AS	-	n.t.
Békéscsaba (ANI)	ANI-3	ovine	III	AS	-	n.t.
Békéscsaba (ANI)	ANI-4	ovine	III	AS	-	n.t.
Békéscsaba (ANI)	ANI-5	ovine	III	AS	-	n.t.
Békéscsaba (ANI)	ANI-6	ovine	III	AS	-	n.t.
Békéscsaba (ANI)	ANI-7	ovine	III	AS	-	n.t.
Békéscsaba (ANI)	ANI-8	ovine	III	AS	-	n.t.
Békéscsaba (ANI)	ANI-9	ovine	III	AS	-	n.t.
Békéscsaba (ANI)	ANI-10	ovine	III	AS	-	n.t.
Békéscsaba (ANI)	ANI-11	ovine	III	AS	-	n.t.
Békéscsaba (ANI)	ANI-12	ovine	III	AS	-	n.t.
Aranyosgadány (AGK)	**AGK-1**	goat	III	AS	+	-
Aranyosgadány (AGK)	AGK-2	goat	III	AS	-	n.t.
Aranyosgadány (AGK)	AGK-3	goat	III	AS	-	n.t.
Aranyosgadány (AGK)	AGK-4	goat	III	AS	-	n.t.
Aranyosgadány (AGK)	AGK-5	goat	III	AS	-	n.t.
Aranyosgadány (AGK)	**AGK-6**	goat	III	AS	+	-
Aranyosgadány (AGK)	AGK-7	goat	III	AS	-	n.t.
Aranyosgadány (AGK)	AGK-8	goat	III	AS	-	n.t.
Aranyosgadány (AGK)	**AGK-9**	goat	II	AS	+	-
Aranyosgadány (AGK)	AGK-10	goat	II	AS	-	n.t.
Aranyosgadány (AGK)	AGK-11	goat	II	AS	-	n.t.
Aranyosgadány (AGK)	**AGK-12**	goat	II	AS	+	-
Aranyosgadány (AGK)	**AGK-13**	goat	II	GE	+	-
Aranyosgadány (AGK)	**AGK-14***	goat	II	AS	+	+
Aranyosgadány (AGK)	**AGK-15**	goat	II	AS	+	-
Aranyosgadány (AGK)	**AGK-16**	goat	II	GE	+	+
Győrszentiván (KT)	KT-FI-1	goat	III	AS	-	n.t.
Győrszentiván (KT)	KT-FI-2	goat	III	AS	-	n.t.
Győrszentiván (KT)	KT-FI-3	goat	III	AS	-	n.t.
Győrszentiván (KT)	KT-FI-4	goat	III	AS	-	n.t.
Győrszentiván (KT)	KT-FI-5	goat	III	AS	-	n.t.
Győrszentiván (KT)	KT-FII-1	goat	III	AS	-	n.t.
Győrszentiván (KT)	KT-FII-2	goat	III	AS	-	n.t.
Győrszentiván (KT)	KT-FII-3	goat	III	AS	-	n.t.

Győrszentiván (KT)	KT-FII-4	goat	III	AS	-	n.t.
Győrszentiván (KT)	KT-FII-5	goat	III	AS	-	n.t.
Győrszentiván (KT)	**KT-G-1**	goat	II	GE	+	+
Győrszentiván (KT)	KT-G-2	goat	II	GE	-	n.t.
Győrszentiván (KT)	KT-G-3	goat	II	GE	-	n.t.
Győrszentiván (KT)	**KT-G-4**	goat	II	GE	+	-
Győrszentiván (KT)	KT-G-5	goat	II	GE	-	n.t.
Győrszentiván (KT)	**KT-G-6**	goat	II	AS	+	-
Győrszentiván (KT)	**KT-G-7**	goat	II	AS	+	+
Győrszentiván (KT)	KT-G-8	goat	II	AS	-	n.t.
Győrszentiván (KT)	**KT-G-9**	goat	II	AS	+	+
Győrszentiván (KT)	KT-G-10	goat	II	AS	-	n.t.
Győrszentiván (KT)	KT-FG-2	goat	I	AS	-	n.t.
Győrszentiván (KT)	KT-FG-3	goat	I	GE	-	n.t.
Győrszentiván (KT)	KT-FG-4	goat	I	AS	-	n.t.
Győrszentiván (KT)	KT-FG-5	goat	I	GE	-	n.t.
Győrszentiván (KT)	KT-FG-6	goat	I	AS	-	n.t.
Győrszentiván (KT)	KT-FG-7	goat	I	GE	-	n.t.
Győrszentiván (KT)	KT-FG-8	goat	I	AS	-	n.t.
Győrszentiván (KT)	KT-FG-9	goat	I	GE	-	n.t.
Győrszentiván (KT)	KT-FG-10	goat	I	AS	-	n.t.
Nagyhegy (NH)	NH-1	goat	III	AS	-	n.t.
Nagyhegy (NH)	**NH-2**	goat	III	AS	+	+
Nagyhegy (NH)	NH-3	goat	III	AS	-	n.t.
Nagyhegy (NH)	**NH-4**	goat	III	AS	+	+
Nagyhegy (NH)	NH-5	goat	III	AS	-	n.t.
Rudabánya (K)	K-1	goat	II	AS	-	n.t.
Rudabánya (K)	K-2	goat	II	AS	-	n.t.
Rudabánya (K)	K-3	goat	II	AS	-	n.t.
Rudabánya (K)	**K-4**	goat	II	AS	+	-
Rudabánya (K)	K-5	goat	II	AS	-	n.t.
Rudabánya (K)	K-6	goat	II	AS	-	n.t.
Rudabánya (K)	K-7	goat	II	AS	-	n.t.
Rudabánya (K)	K-8	goat	II	AS	-	n.t.
Rudabánya (K)	K-9	goat	II	AS	-	n.t.
Rudabánya (K)	K-10	goat	II	AS	-	n.t.
Rudabánya (K)	K-11	goat	II	AS	-	n.t.
Rudabánya (K)	K-12	goat	II	AS	-	n.t.
Hajdúböszörmény (HB)	HB-7369	cattle	I	AS	-	n.t.
Hajdúböszörmény (HB)	HB-7660	cattle	I	AS	-	n.t.
Hajdúböszörmény (HB)	HB-7373	cattle	I	AS	-	n.t.

Hajdúböszörmény (HB)	HB-7431	cattle	I	GE	-	n.t.
Hajdúböszörmény (HB)	HB-7396	cattle	I	AS	-	n.t.
Hajdúböszörmény (HB)	HB-7693	cattle	I	AS	-	n.t.
Hajdúböszörmény (HB)	HB-7433	cattle	I	AS	-	n.t.
Hajdúböszörmény (HB)	HB-7248	cattle	I	AS	-	n.t.
Hajdúböszörmény (HB)	HB-7615	cattle	I	GE	-	n.t.
Hajdúböszörmény (HB)	HB-7315	cattle	I	AS	-	n.t.
Hajdúböszörmény (HB)	HB-7657	cattle	I	GE	-	n.t.
Hajdúböszörmény (HB)	HB-7500	cattle	I	AS	-	n.t.
Hajdúböszörmény (HB)	HB-7673	cattle	I	GE	-	n.t.
Hajdúböszörmény (HB)	HB-E-1	cattle	III	AS	-	n.t.
Hajdúböszörmény (HB)	HB-7627	cattle	I	AS	-	n.t.
Hajdúböszörmény (HB)	HB-7751	cattle	I	GE	-	n.t.
Hajdúböszörmény (HB)	**HB-8066**	cattle	I	AS	+	-
Hajdúböszörmény (HB)	HB-0303	cattle	I	GE	-	n.t.
Hajdúböszörmény (HB)	HB-7663	cattle	I	AS	-	n.t.
Hajdúböszörmény (HB)	HB-7394	cattle	I	AS	-	n.t.
Hajdúböszörmény (HB)	**HB-P1**	cattle-pool	III	-	+	+
Nyíregyháza (NyH)	NYH-3175	cattle	I	AS	-	n.t.
Nyíregyháza (NyH)	NYH-3254	cattle	I	AS	-	n.t.
Nyíregyháza (NyH)	NYH-3257	cattle	I	AS	-	n.t.
Nyíregyháza (NyH)	NYH-3259	cattle	I	AS	-	n.t.
Nyíregyháza (NyH)	NYH-3256	cattle	I	AS	-	n.t.
Nyíregyháza (NyH)	NYH-GI-1	cattle	II	AS	-	n.t.
Nyíregyháza (NyH)	NYH-GI-2	cattle	II	AS	-	n.t.
Nyíregyháza (NyH)	NYH-GII-1	cattle	II	AS	-	n.t.
Nyíregyháza (NyH)	NYH-GII-2	cattle	II	AS	-	n.t.
Nyíregyháza (NyH)	NYH-GIII-1	cattle	III	AS	-	n.t.
Nyíregyháza (NyH)	NYH-GIII-2	cattle	III	AS	-	n.t.
Derecske (DR)	DR-1	cattle	III	AS	-	n.t.
Derecske (DR)	DR-2	cattle	III	AS	-	n.t.
Derecske (DR)	DR-3	cattle	III	AS	-	n.t.
Derecske (DR)	DR-4	cattle	III	AS	-	n.t.
Derecske (DR)	DR-5	cattle	III	AS	-	n.t.
Derecske (DR)	DR-6	cattle	III	AS	-	n.t.
Derecske (DR)	DR-7	cattle	III	AS	-	n.t.
Derecske (DR)	DR-8	cattle	III	AS	-	n.t.
Derecske (DR)	DR-9	cattle	III	AS	-	n.t.
Derecske (DR)	DR-10	cattle	III	AS	-	n.t.
Derecske (DR)	DR-11	cattle	III	AS	-	n.t.
Derecske (DR)	DR-B-1	cattle	I	AS	-	n.t.

Derecske (DR)	DR-B-2	cattle	I	AS	-	n.t.
Derecske (DR)	DR-B-3	cattle	I	AS	-	n.t.
Derecske (DR)	DR-B-4	cattle	II	AS	-	n.t.
Tiszavasvári (TiV)	TiV-001	cattle	I	AS	-	n.t.
Tiszavasvári (TiV)	TiV-002	cattle	I	AS	-	n.t.
Tiszavasvári (TiV)	TiV-003	cattle	I	AS	-	n.t.
Tiszavasvári (TiV)	TiV-004	cattle	I	AS	-	n.t.
Tiszavasvári (TiV)	TiV-005	cattle	I	AS	-	n.t.
Tiszavasvári (TiV)	TiV-GI-1	cattle	II	AS	-	n.t.
Tiszavasvári (TiV)	TiV-GI-2	cattle	II	AS	-	n.t.
Tiszavasvári (TiV)	TiV-GI-3	cattle	II	AS	-	n.t.
Tiszavasvári (TiV)	TiV-GII-1	cattle	II	AS	-	n.t.
Tiszavasvári (TiV)	TiV-GII-2	cattle	II	AS	-	n.t.
Tiszavasvári (TiV)	TiV-GIII-1	cattle	II	AS	-	n.t.
Tiszavasvári (TiV)	TiV-GIII-2	cattle	II	AS	-	n.t.
Tiszavasvári (TiV)	TiV-F1	cattle	III	AS	-	n.t.
Tiszavasvári (TiV)	TiV-007	cattle	I	AS	-	n.t.
Tiszavasvári (TiV)	TiV-6379	cattle	I	AS	-	n.t.
Tiszavasvári (TiV)	TiV-0577	cattle	I	AS	-	n.t.
Bonyhád (BH)	BH-0001	cattle	I	AS	-	n.t.
Bonyhád (BH)	BH-9812	cattle	I	AS	-	n.t.
Bonyhád (BH)	BH-9852	cattle	I	AS	-	n.t.
Bonyhád (BH)	BH-9854	cattle	I	AS	-	n.t.
Bonyhád (BH)	BH-9858	cattle	I	AS	-	n.t.
Bonyhád (BH)	BH-9861	cattle	I	AS	-	n.t.
Bonyhád (BH)	BH-9865	cattle	I	AS	-	n.t.
Bonyhád (BH)	BH-9878	cattle	I	AS	-	n.t.
Bonyhád (BH)	BH-9901	cattle	I	AS	-	n.t.
Bonyhád (BH)	BH-9955	cattle	I	AS	-	n.t.
Bonyhád (BH)	BH-9963	cattle	I	AS	-	n.t.
Bonyhád (BH)	BH-9971	cattle	I	AS	-	n.t.
Bonyhád (BH)	BH-9974	cattle	I	AS	-	n.t.
Bonyhád (BH)	BH-9989	cattle	I	AS	-	n.t.
Bonyhád (BH)	BH-9993	cattle	I	AS	-	n.t.
Bonyhád (BH)	BH-9994	cattle	I	AS	-	n.t.
Tevel (TV)	**TV-9682***	cattle	I	AS	+	+
Tevel (TV)	**TV-9686**	cattle	I	AS	+	+
Tevel (TV)	TV-9703	cattle	I	AS	-	n.t.
Tevel (TV)	TV-9728	cattle	I	AS	-	n.t.
Tevel (TV)	TV-9733	cattle	I	AS	-	n.t.
Tevel (TV)	TV-9736	cattle	I	AS	-	n.t.

Tevel (TV)	TV-9738	cattle	I	AS	-	n.t.
Tevel (TV)	TV-9743	cattle	I	AS	-	n.t.
Tevel (TV)	TV-9746	cattle	I	AS	-	n.t.
Tevel (TV)	TV-9760	cattle	I	AS	-	n.t.
Tevel (TV)	TV-9764	cattle	I	AS	-	n.t.
Tevel (TV)	TV-9772	cattle	I	AS	-	n.t.
Tevel (TV)	TV-9774	cattle	I	AS	-	n.t.
Tevel (TV)	TV-9779	cattle	I	AS	-	n.t.
Tevel (TV)	TV-9785	cattle	I	AS	-	n.t.
Tevel (TV)	TV-9787	cattle	I	AS	-	n.t.
Tevel (TV)	TV-9789	cattle	I	AS	-	n.t.
Egyházasfalu (EF)	EF-1	swine	II	AS	-	n.t.
Egyházasfalu (EF)	EF-2	swine	II	AS	-	n.t.
Egyházasfalu (EF)	EF-3	swine	II	AS	-	n.t.
Egyházasfalu (EF)	EF-4	swine	II	AS	-	n.t.
Egyházasfalu (EF)	EF-5	swine	II	AS	-	n.t.
Egyházasfalu (EF)	EF-6	swine	II	AS	-	n.t.
Egyházasfalu (EF)	EF-7	swine	II	AS	-	n.t.
Egyházasfalu (EF)	EF-8	swine	II	AS	-	n.t.
Egyházasfalu (EF)	EF-9	swine	II	AS	-	n.t.
Egyházasfalu (EF)	EF-10	swine	II	AS	-	n.t.
Szigetvár (SzV)	SzV-1018-1	swine	I	AS	-	n.t.
Szigetvár (SzV)	SzV-1018-2	swine	I	AS	-	n.t.
Szigetvár (SzV)	SzV-1018-3	swine	I	AS	-	n.t.
Szigetvár (SzV)	SzV-1018-4	swine	I	AS	-	n.t.
Szigetvár (SzV)	SzV-1018-5	swine	I	AS	-	n.t.
Szigetvár (SzV)	SzV-1018-6	swine	I	AS	-	n.t.
Szigetvár (SzV)	SzV-1018-7	swine	I	AS	-	n.t.
Szigetvár (SzV)	SzV-1018-8	swine	I	AS	-	n.t.
Szigetvár (SzV)	SzV-1018-9	swine	I	AS	-	n.t.
Szigetvár (SzV)	SzV-1018-10	swine	I	AS	-	n.t.
Szigetvár (SzV)	SzV-1018-11	swine	I	AS	-	n.t.
Szigetvár (SzV)	SzV-1018-12	swine	I	AS	-	n.t.
Szigetvár (SzV)	SzV-1018-13	swine	I	AS	-	n.t.
Szigetvár (SzV)	SzV-1018-14	swine	I	AS	-	n.t.
Szigetvár (SzV)	SzV-1018-15	swine	I	AS	-	n.t.
Szigetvár (SzV)	SzV-1018-16	swine	I	AS	-	n.t.
Szigetvár (SzV)	SzV-1018-17	swine	I	AS	-	n.t.
Szigetvár (SzV)	SzV-1018-18	swine	I	AS	-	n.t.
Szigetvár (SzV)	SzV-1018-19	swine	I	AS	-	n.t.
Szigetvár (SzV)	SzV-1018-20	swine	I	AS	-	n.t.

Szigetvár (SzV)	SzV-1018-21	swine	I	AS	-	n.t.
Szigetvár (SzV)	SzV-1018-22	swine	I	AS	-	n.t.
Szigetvár (SzV)	SzV-1018-23	swine	I	AS	-	n.t.
Szigetvár (SzV)	SzV-1018-24	swine	I	AS	-	n.t.
Orosháza (OR)	OR-1	swine	I	AS	-	n.t.
Orosháza (OR)	OR-2	swine	I	AS	-	n.t.
Orosháza (OR)	OR-3	swine	I	AS	-	n.t.
Orosháza (OR)	OR-4	swine	I	AS	-	n.t.
Orosháza (OR)	OR-5	swine	I	AS	-	n.t.
Orosháza (OR)	OR-6	swine	I	AS	-	n.t.
Orosháza (OR)	OR-7	swine	I	AS	-	n.t.
Orosháza (OR)	OR-8	swine	I	AS	-	n.t.
Orosháza (OR)	OR-9	swine	I	AS	-	n.t.
Somogysárd (SS)	SS-1-A	rabbit	I	AS	-	n.t.
Somogysárd (SS)	SS-1-B	rabbit	I	AS	-	n.t.
Somogysárd (SS)	SS-1-C	rabbit	I	AS	-	n.t.
Somogysárd (SS)	SS-1-D	rabbit	I	AS	-	n.t.
Somogysárd (SS)	SS-1-E	rabbit	I	AS	-	n.t.
Somogysárd (SS)	SS-1-F	rabbit	I	AS	-	n.t.
Somogysárd (SS)	SS-1-G	rabbit	I	AS	-	n.t.
Somogysárd (SS)	SS-1-H	rabbit	I	AS	-	n.t.
Somogysárd (SS)	SS-1-I	rabbit	I	AS	-	n.t.
Somogysárd (SS)	SS-1-J	rabbit	I	AS	-	n.t.
Somogysárd (SS)	SS-1-K	rabbit	I	AS	-	n.t.
Somogysárd (SS)	SS-1-L	rabbit	I	AS	-	n.t.
Somogysárd (SS)	SS-1-M	rabbit	I	AS	-	n.t.
Somogysárd (SS)	SS-2-A	rabbit	II	AS	-	n.t.
Somogysárd (SS)	SS-2-B	rabbit	II	AS	-	n.t.
Somogysárd (SS)	SS-2-C	rabbit	II	AS	-	n.t.
Somogysárd (SS)	SS-2-D	rabbit	II	AS	-	n.t.
Somogysárd (SS)	SS-2-E	rabbit	III	AS	-	n.t.
Somogysárd (SS)	SS-2-F	rabbit	III	AS	-	n.t.
Somogysárd (SS)	SS-2-G	rabbit	III	AS	-	n.t.
Somogysárd (SS)	SS-2-H	rabbit	III	AS	-	n.t.

Table S2: List of oligonucleotide primers used for screening and typing reactions of this study.

Reaction type	Target virus(es)	Primer name	5' - 3' sequence	5' Position	Product size (bp)
screening	*Boosepivirus C*	OvEncePV-Screen-R	GGC CAC TCR CCA TAR GCA AC	387[a]	640
		OvEncePV-Screen-F	AGC GCC YTG AAT GCG GCT AA	1026[a]	
screening	ovipi-, gopi- & bopiviruses	HBG-3D-Screen-R	GTC CAT GAC AGG GTG AAT CA	7110[b]	627
		HBG-3D-Screen-F	CTG RGC AAG TTC ACC AAC AA	6484[b]	
typing	ovipivirus	Ovipi-VP1-Fgen	TCT GCA ACC GAC TWC CGC TA	2140[b]	1169
		Ovipi-VP1-Rgen	TTG GWY TCA ATG TCA CCA CC	3308[b]	
typing	bopivirus Hun/A1	Bopi-VP1-Fgen	CTW GTS TGG GAC ATT GG	2256[c]	1180
		Bopi-VP1-Rgen	GTC CAG AAA GGR YGC GG	3436[c]	
typing	ovipi-& gopiviruses	HoGo-VP1-Fgen	CTY ATC TGG GAC ATT GG	2086[b]	1135
		HoGo-VP1-Rgen	CAG AAW GGT CTG GGA CA	3221[b]	
typing	gopivirus	Gopi-VP1-Falt	CGT CCG ACA TGA TCG AYG CT	2449[d]	907
		HBG-NPGP-Rgen	CCA GGG TTG GWY TCR ATG TC	3355[d]	

Note that 5′ positions of the primers apply to: [a] Boosepivirus C (LR216006); [b] ovipivirus strain ovine/TB14/2010-HUN; [c] bopivirus/Hun strain bovine/TV-9682/2019-HUN; [d] gopivirus strain goat/AGK14/2020-HUN.
